# Mechanisms influencing the factors of urban built environments and coronavirus disease 2019 at macroscopic and microscopic scales: The role of cities

**DOI:** 10.3389/fpubh.2023.1137489

**Published:** 2023-02-28

**Authors:** Longhao Zhang, Xin Han, Jun Wu, Lei Wang

**Affiliations:** ^1^School of Architecture, Tianjin Chengjian University, Tianjin, China; ^2^Department of Landscape Architecture, Kyungpook National University, Daegu, Republic of Korea; ^3^School of Architecture, Tianjin University, Tianjin, China

**Keywords:** COVID-19, urban built environment, relevance, street view images, computer vision, deep learning

## Abstract

In late 2019, the coronavirus disease 2019 (COVID-19) pandemic soundlessly slinked in and swept the world, exerting a tremendous impact on lifestyles. This study investigated changes in the infection rates of COVID-19 and the urban built environment in 45 areas in Manhattan, New York, and the relationship between the factors of the urban built environment and COVID-19. COVID-19 was used as the outcome variable, which represents the situation under normal conditions vs. non-pharmacological intervention (NPI), to analyze the macroscopic (macro) and microscopic (micro) factors of the urban built environment. Computer vision was introduced to quantify the material space of urban places from street-level panoramic images of the urban streetscape. The study then extracted the microscopic factors of the urban built environment. The micro factors were composed of two parts. The first was the urban level, which was composed of urban buildings, Panoramic View Green View Index, roads, the sky, and buildings (walls). The second was the streets' green structure, which consisted of macrophanerophyte, bush, and grass. The macro factors comprised population density, traffic, and points of interest. This study analyzed correlations from multiple levels using linear regression models. It also effectively explored the relationship between the urban built environment and COVID-19 transmission and the mechanism of its influence from multiple perspectives.

## 1. Introduction

Novel pneumonia caused by coronavirus 2019 (COVID-19), which leads to severe acute respiratory syndrome, has been raging worldwide for nearly 3 years since December 2019 (1). The speed of transmission of the virus, its infectiousness, and the number of mutations have been the most unprecedented in human medical history. Large cities and metropolitan areas have been the areas most affected by the spread of the virus, exacerbated by the areas' dense population distribution (2). To strictly control the rate of COVID-19 transmission and to reduce the rates of infection and deaths, countries have adopted non-pharmaceutical interventions (NPIs) including urban lockdown, home isolation, controlled social distancing, and travel restrictions (3, 4). Ever since it reared its ugly head, COVID-19 has attracted substantial attention from the global community, and various studies on COVID-19 have emerged accordingly. A majority of the studies have focused on the related factors of sociodemographics and the urban built environment. The results vary from two different research perspectives.

From the sociodemographic perspective, the risk of COVID-19 infection is much higher for the elderly and children than it is for young and middle-aged adults (5–7). In addition, the degree of economic development across regions may exert an impact on the transmission rate of COVID-19 (8, 9). For example, the availability of health insurance has been highly correlated with the spread of COVID-19 (10). Low-income areas, especially older communities with low levels of income, have been more susceptible to COVID-19 infection (11, 12), all of which have been related to regional economic development. Using logistic regression models, several studies have demonstrated that levels of regional literacy are also associated with the prevalence of COVID-19 (13). Other factors have also been correlated with COVID-19 transmission, such as blood type, respiratory disease, and chronic diseases. Additionally, personal habits have been associated with COVID-19 transmission (14, 15). The strict implementation of NPI against COVID-19 has proven effective in mitigating the spread of the virus (16, 17). In the post-pandemic era, the patterns of behavior in daily life have changed due to reliable NPIs implemented by governments (18). Under the influence of NPIs, the range of activities of urban residents has been significantly reduced, thereby rendering them increasingly dependent largely on the surroundings of their homes, the natural urban environment, and the built urban environment (19). The surroundings of urban homes and the built environment have exerted a direct effect on the physical and mental health of urban residents (20). Simultaneously, low-density neighborhoods, large homes, developed urban residential surroundings and infrastructure, rich urban greenery, and large urban green spaces can greatly enhance the life satisfaction and wellbeing of residents under COVID-19 NPIs (21).

From the perspective of the urban built environment, the impact of the urban built environment on COVID-19 is extremely important in addition to socio-demographic factors, which has been confirmed by many studies. The relationship between COVID-19 transmission and population density is relatively controversial. Previous studies demonstrate that the incidence and transmission of COVID-19 are higher in densely populated areas with high population contact (22). A comparison of the results of linear regression models from 182 countries points to a positive association between population density and COVID-19 transmission (23). In contrast, the results of structural equation modeling at the city level illustrate that population density is negatively associated with COVID-19 transmission in Tehran (24). This result is interesting, where a few studies argue that urban population density is non-significantly correlated with the spread of COVID-19 (25). The relationship between urban population density and the transmission rate of COVID-19 is complex. Thus, the various responses of governments and urban residents to the pandemic across nations may lead to different results, which are reasonably explained by the findings of Hamidi et al. (26) in the United States, Lin et al. (27) in China, and Boterman (28) in the Netherlands. Meanwhile, the relationship between urban building density and COVID-19 lacks elucidation and is subject to a certain degree of controversy (29). A few studies demonstrate that no correlation exists between building density and COVID-19 after omitting certain confounding factors (28). During the COVID-19 pandemic and under government NPIs, the wellbeing of residents living in high-density areas was negatively correlated with living density due to changes in the scope of life and lifestyle behaviors. However, this compact urban form leads to relatively easy access to urban healthcare resources, which could improve the health status of residents (18, 30).

From the perspective of the urban built environment alone, different factors in the urban built environment may have an impact on the spread and transmission of COVID-19 during an epidemic pandemic. For example, public transportation (31) and points of interest (POI) (32), among others, are generally considered to exhibit a positive association with COVID-19 transmission. When the outbreak was in its emergent stage, public transportation was considered the main method of COVID-19 transmission. Therefore, many governments advised urban residents to avoid public transportation as much as possible while introducing corresponding NPIs and limiting the range of activities of residents. In addition, they frequently urged urban residents to use multiple modes of transportation, such as self-driving, walking, and cycling (33). The results of analyses using multiscale geographically weighted regression suggested that the high availability of medical resources around a community could effectively inhibit the spread of COVID-19 (7). Through structural equation modeling and categorical regression modeling, other analyses demonstrated high-quality housing and high-quality green space as being negatively associated with the spread of COVID-19 (10, 34). Green spaces around large residential areas exerted an inhibitory effect on the deterioration of urban health and wellbeing due to COVID-19 (30). Notably, the risk of infection and transmission rates were high for neighborhoods with high levels of community convenience (35). Integrating all patients with COVID-19 into high-grade urban hospitals is unrealistic because hospital capacity is far from adequate for treating such a large number of patients at the burgeoning stage of a pandemic such as COVID-19. Moreover, the risk of collapse of urban public healthcare is prevalent as demonstrated by the collapse of public healthcare to varying degrees in various countries during the COVID-19 outbreak. Therefore, a community-level system for identifying and isolating individuals with infection is essential to the response to COVID-19 (36).

In summary, several questions can be elicited from the influence of the urban built environment on the spread of COVID-19: (I) how the macroscopic urban built environment and the microscopic urban built environment have an impact on the spread of COVID-19 in the urban built environment; and (II) what is the impact of the macroscopic urban built environment and the microscopic built environment on the incidence and lethality of COVID-19. However, most of the community-level studies at this stage have used administrative boundaries to delineate the selection of variables, and the disadvantage of this method of variable selection is that it does not reflect the actual activities of residents. In this regard, Li et al. used structural equation modeling to reveal the relationship between commercial vitality and transportation infrastructure on the increase in the number of confirmed cases, and innovatively used buffer zones to extract urban built environment factors around confirmed cases (37). Wang et al. used walking circles at different times to investigate the correlation between urban built environment and community level spatial distribution (38). By extracting the established environmental factors in both spatial dimensions and examining the correlation between these factors and the prevalence of COVID-19, the issue of the transmission mechanism of COVID-19 before and after the implementation of community-level NPI measures was then analyzed. Studies at this stage ignore the lack of multi-level studies on the mechanisms of the urban micro-built environment influencing the spread of COVID-19. Whether the urban street green environment and urban street spatial quality have an impact on the spread of COVID-19 has not been explored, and the impact of urban built environment on the long-term trend and overall trend of COVID-19 has not been considered comprehensively. In this study, based on the study of the influence mechanism between the macroscopic built environment and COVID-19, the influence mechanism between the microscopic built environment and COVID-19 was considered at multiple levels using Google Street View panoramic street view images. The impact of urban built environment on the long-term trend and overall trend of COVID-19 is investigated using multiple variables, and the influence mechanism of urban built environment on COVID-19 is examined at multiple levels (macro level and micro level) and multiple dimensions (time dimension). The results of the study can provide a basis and reference for governmental decision makers to formulate more reasonable NPI policies to slow down the spread of COVID-19 during pandemic periods. The results of the study may provide a reference solution to control the spread and spread of the virus, and the results may provide effective recommendations to contain potential respiratory disease outbreaks.

## 2. Data

### 2.1. Research region

New York City is considered the first epicenter of the COVID-19 outbreak in the United States. It has a population of ~8.51 million (as of 2017) and an area of ~1,214 km^2^ (including the sea). With an average of 28 people per square mile, New York City is the main international maritime, airport, and financial metropolis of the United States and has five boroughs under its jurisdiction, namely, Brooklyn, Queens, Manhattan, the Bronx, and Staten Island.

**Figure 1:**
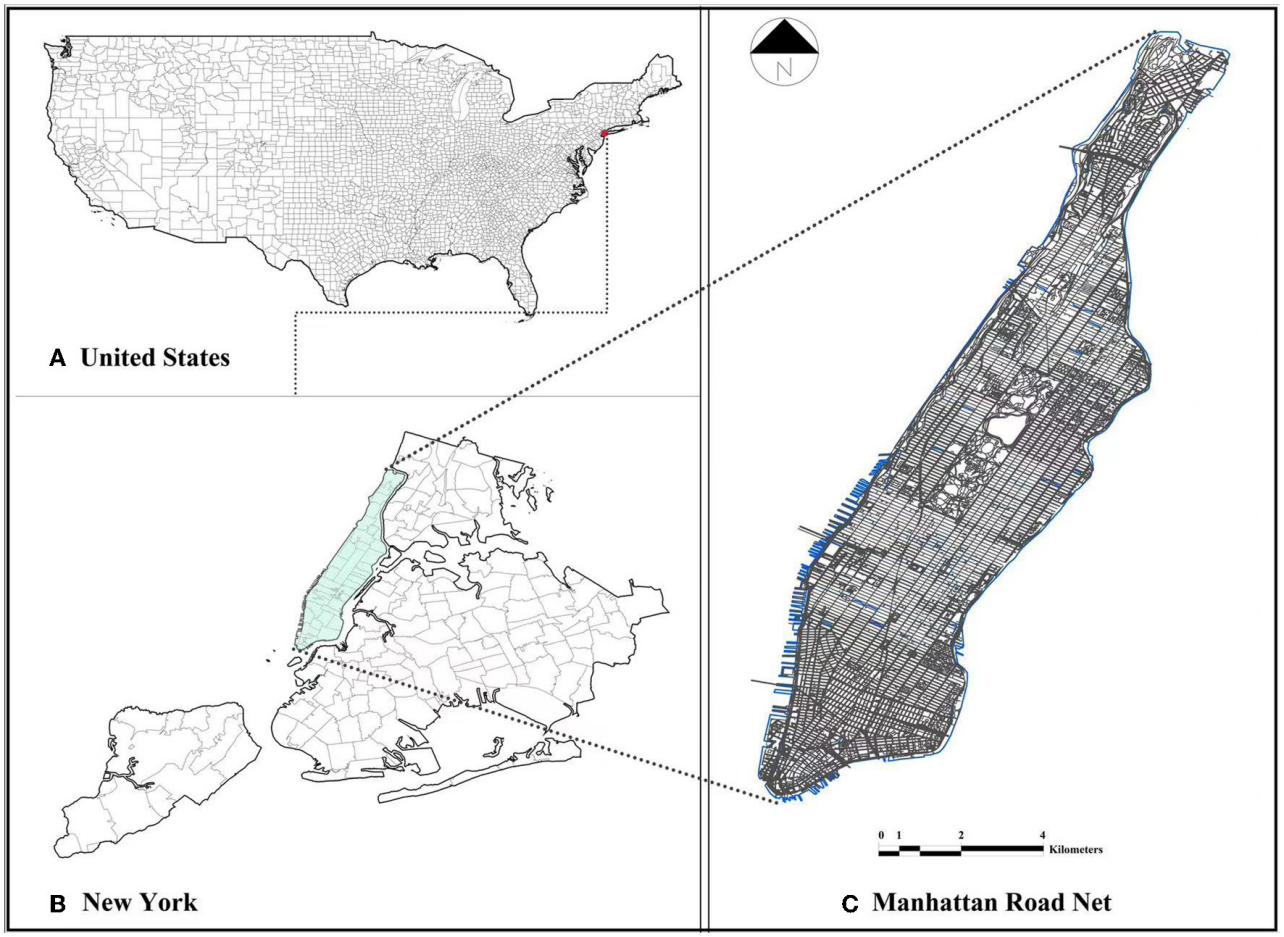
Study area: **(A)** Map of the United States; **(B)** New York County; and **(C)** Manhattan road network.

Manhattan is the most densely populated and smallest of the five boroughs of New York City, which translates to a very high population and housing density when compared with those of other boroughs in New York City. Manhattan is described as the economic and cultural center of the United States and is home to New York's central business district, which houses the headquarters of most Fortune 500 companies and the headquarters of the United Nations. Thus, the nearly 50 million tourists who visit New York City each year significantly contribute to the risk of COVID-19 transmission and routes of transmission. Figure 1 describes the study area and its road network. As the center of the metropolitan area, a major outbreak of COVID-19 is likely to spread rapidly to other areas of the metropolis and continue to expand outward. Thus, understanding the relationship between the spread of COVID-19 and the factors of the urban built environment is an important aspect for urban decision-makers in mitigating the spread of the disease and in developing openness measures.

### 2.2. Google street view images and dataset

#### 2.2.1. Google street view images

**Figure 2:**
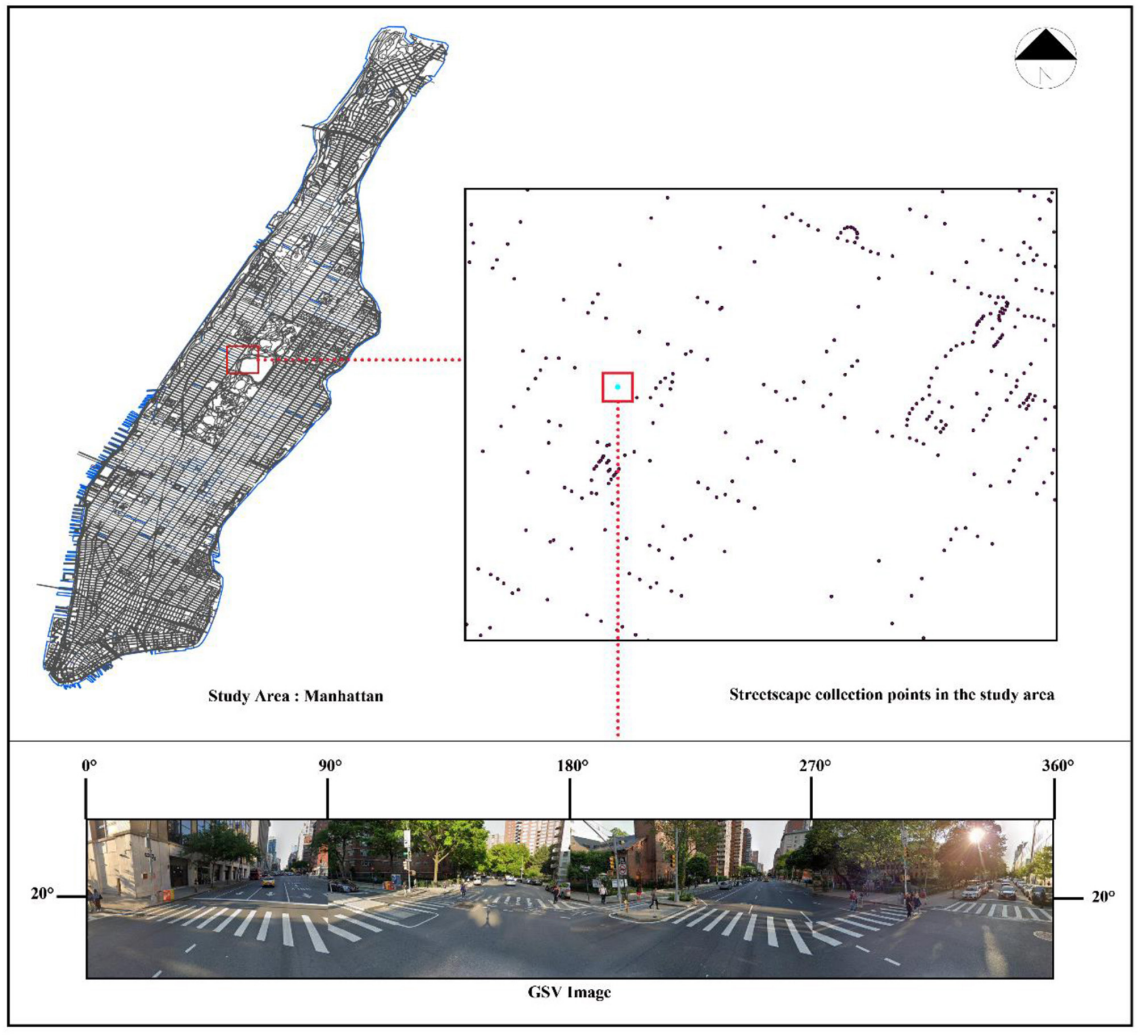
Demonstration of the acquisition of GSV images.

The study obtained urban street panorama images from Google Maps to reflect the physical characteristics of the urban environment. Factors related to the urban environment were extracted from these images as evaluation indexes of the urban environment. To improve the representativeness of the physical features and environmental factors of the urban environment, the study created a collection point for every 100 m on all urban roads in the study area. A total of 67,025 collection points were set up to collect images with each collection point having one image based on a 90° view. Moreover, the study collected four images for each collection point to synthesize the panoramic streetscape images, which reached 268,100 images. The images were cleaned according to the availability of data, and all images were collected from Google Street View (GSV) to analyze the physical characteristics of the city and extract the factors of the urban environment. By appropriately establishing the parameters for image retrieval, the images captured both sides and frontal images of the street. This image acquisition covered all roads in Manhattan. Figure 2 provides a demonstration of the acquisition of the GSV images.

#### 2.2.2. Dataset for training the neural network model

The study used datasets from Cityscape, ADE20K, and S-S-G-S to train the neural network model, a dataset open to researchers at the Mercedes-Benz R&D Center and Darmstadt University of Technology and published in the 2016 Clean Vehicle Rebate Project. The dataset was collected from 50 cities in Germany and nearby countries, including street scenes in spring, summer, and autumn. Different annotators with 96 and 98% pixel consistencies repeatedly annotated the 30 selected data after omitting categories that could be annotated as unclear. The drawback was that the segmentation dataset contained 33 classes, whereas the validation dataset was composed of only 19 semantic segmentation classes because the data volume of a few classes was very sparse. The ADE20K dataset is intended for Scene Understanding, which was opened by the Massachusetts Institute of Technology (MIT) in 2016 and can be used, for instance, in semantics and part segmentation. Using image information for Scene Understanding and parsing, the dataset consists of 27,000 images from Scene Understanding (an open dataset released by Princeton University in 2010) and Places (an open dataset by MIT released in 2014). The ADE20K contains more than 3,000 object classes, which greatly compensates for the shortcomings of the Cityscape dataset. The S-S-G-S dataset was constructed by Zhang et al. (39) in 2022 and is mainly used for the analysis of urban vegetation communities. A neural network model trained using this dataset can classify and visualize the structure of urban street vegetation communities. S-S-G-S differs from the Cityscape and ADE20K datasets in that it is directed toward the analysis of urban greenery. The study mainly used the trained DeepLabV3+ neural network model to extract urban features at the micro level.

### 2.3. COVID-19 dataset

The first confirmed case of COVID-19 was reported in Manhattan on March 1, 2020. At the time, the number of confirmed cases of COVID-19 in the entire United States was only 76. However, as of March 25, 2020, the number of confirmed COVID-19 cases in the United States spiked to 69,008, and the number of deaths reached 1,045, such that COVID-19 rampaged through the country at a rate of 10,000 per day for three consecutive days. However, according to the Centers for Disease Control and Prevention, nearly 50% of all confirmed cases in the United States as of March 25, 2020, are in New York State, which establishes it as the epicenter of the outbreak. Out of the 33,006 cases diagnosed in New York State, 20,011 were derived from New York City. Over time and with the introduction of various restrictive policies and concerted national efforts to combat the outbreak, the spread of COVID-19 decelerated. Moreover, the outbreak appeared to be moving in a positive direction with the advent of COVID-19 vaccines.

Moving forward to late December 2021, a variant of COVID-19 (omicron) is once again ravaging New York State with a record-breaking 21,908 cases detected in New York State on December 18, 2021, alone. Moreover, an alarming spike in cases was noted in several highly vaccinated neighborhoods in Manhattan. With 7-day positivity rates exceeding 10% in more than 10 areas of New York City from December 10 to 16, 2021, Manhattan, once again, clearly became a hotbed of COVID-19 transmission. A total of 790.87 cases were identified per 100,000 people, and an extremely alarming rate was noted in specific Manhattan neighborhoods as of December 24, 2021. Greenwich Village and SoHo reported 2,850 confirmed cases per 100,000 people, and Chelsea reached 2,400 confirmed cases per 100,000 people. Nevertheless, no pandemic hotspot in the nation could compare to the dire outbreak in Greenwich Village.

The COVID-19 case data in the study were derived from the publication by NYC Health (https://www1.nyc.gov/site/doh/index.page), which included cumulative totals since the COVID-19 outbreak in New York City. The Department of Health (DOH) defined the first case of COVID-19 as the one confirmed on February 29, 2020. In addition, the DOH recommended the avoidance of interpreting the daily changes in these files as 1-day data due to the discrepancy between the date of the event and the date of reporting. The internal division of the study area was divided according to the Modified ZIP Code Tabulation Areas (MODZCTA).

NYC Health uses MODZCTA to report information according to geographic location. However, several issues emerge when mapping data reported based on ZIP codes because they do not designate a single area but a collection of points that compose the route of mail delivery. Moreover, a few buildings and non-residential areas were frequently assigned unique ZIP codes. To address these issues, the DOH uses ZIP Code Tabulation Areas (ZCTA) to convert ZIP codes into area units. The United States Census Bureau developed ZCTA geography to map data reported according to ZIP codes using ZCTA. MODZCTA geography combines census blocks with small populations to provide stable estimates of population size for rate calculations. The visualization is available on the website of NYC Health, which also open-sources the case data (https://github.com/nychealth/coronavirus-data#geography-zip-codes-and-zctas). In this manner, accessing appropriate data is easy for researchers.

This study used Manhattan, New York in the United States as the study area and created a fishing network according to the 68 zones of MODZCTA to compare the mechanisms between the factors of the urban built environment and COVID-19 transmission in different zones and to investigate the reason Manhattan became the center of the pandemic many times during the outbreak.

## 3. Methodology

### 3.1. Outcome variable: COVID-19

The outcome variables of the study were the number of confirmed and suspected cases of COVID-19 [COVID_CASE_COUNT (CCC)] and the incidence of confirmed and suspected cases of COVID-19 per 100,000 people [COVID_CASE_RATE (CCR)] in Manhattan, New York, United States. The difference between CCC and CCR is that CCR is a longer-term trend than CCC, and the relationship between the factors of urban built environment and COVID-19 under NPIs can be determined by comparing with CCC.

**Figure 3:**
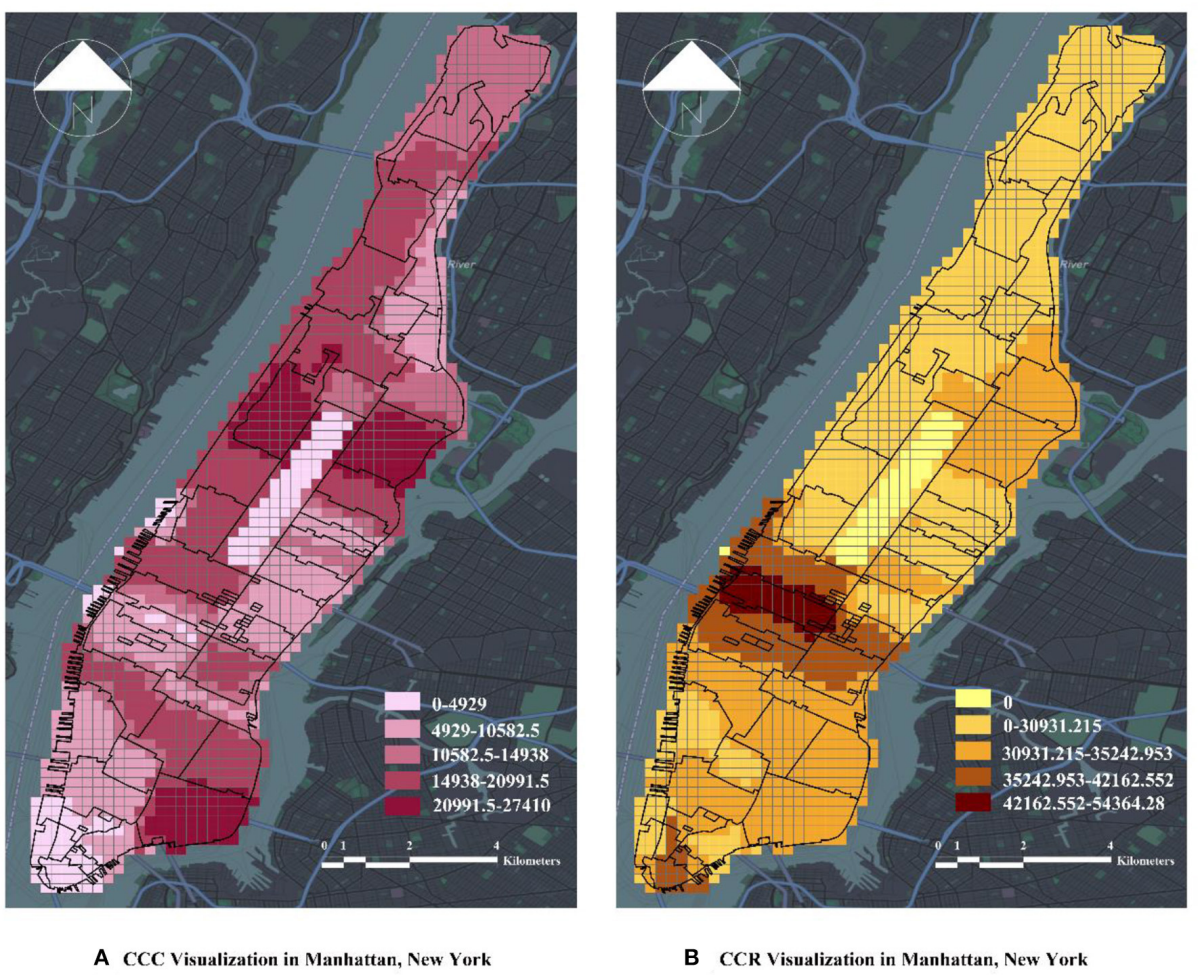
Visualization of CCC and CCR in the study area. **(A)** CCC Visualization in Manhattan, New York. **(B)** CCR Visualization in Manhattan, New York.

According to MODZCTA, the Manhattan area of New York City, United States, was divided into 68 areas. After data filtering, the study identified 45 valid areas, and a fishing net was generated within the study area for a total of 1,551 grids. These grids will be used for analysis and spatial cells. The study calculated the CCC and CCR of the 45 independent areas and averaged them according to the fishing nets to reflect the overall number of cases in Manhattan. In addition, by calculating and visualizing the average of the number of valid POIs and environmental factors of the urban streetscape within the grids, the study intends to better establish the relationship of the urban built environment at the macro- and micro-levels to COVID-19. Figure 3 presents the visualization results of CCC and CCR in the study area.

### 3.2. Macro-scale: Factors of urban built environment

The study selected only three aspects, namely, density, diversity, and traffic, from the 5D's model framework (40, 41) for the evaluation of the factors of the built urban environment and the physical characteristics of the city at the macro level. In terms of density, the study used urban population density as an evaluation indicator. In the evaluation index of diversity, the study selected the data on POIs to measure the diversity of the urban environment. A POI consists of fine-grained data that comprehensively reflect accurate information on urban land use. The POI data used in the study were downloaded from OpenStreetMap (OSM) and reclassified according to the basic functions of the city after the data were screened, which included the omission of irrelevant, duplicate, and empty data. The study obtained 16,003 valid entity POIs for Manhattan, which were classified using C·M·E·P·R (Table 1). The C·M·E·P·R classification, as a method of classifying urban POIs on the basis of built-up characteristics, categorizes urban POIs according to urban functions such as commerce, healthcare, education, public services, and entertainment. Moreover, the POIs were classified according to C·M·E·P·R. The valid POIs were mapped to the fishnet grid of the study area, and the entropy score of the POI data per grid was calculated to determine diversity (42), which is calculated as follows:


$$\begin{array}{l} Mix\;Index=-{\sum^{\text{​}}}_{i=\;1}^{n}p_{i}lnp_{i}. \end{array}$$

**Figure 4:**
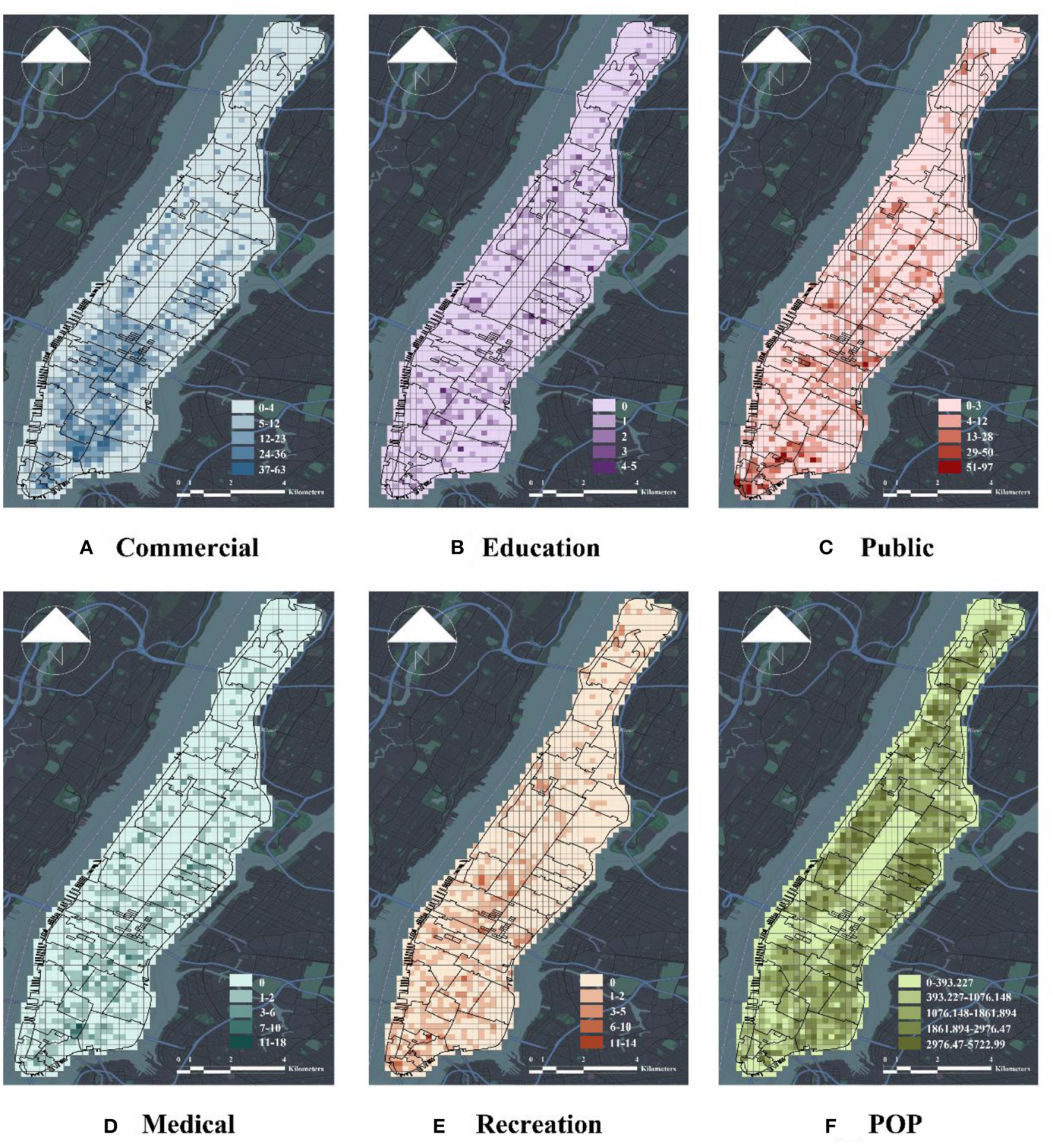
Visualization of macro-scale factors of urban built environment. **(A)** Commercial, **(B)** education, **(C)** public, **(D)** medical, **(E)** recreation, and **(F)** POP.

The formula pi is the proportion of the *i*th type of POI, and *n* is the total number of all POI types in the fishing grid. In turn, it better reflects the influence relationship between the urban built environment and COVID-19. Figure 4 provides the visualization results of macro factors of the urban built environment.

**Table 1:** Classification of POI types according to C·M·E·P·R and corresponding categories in the original OpenStreetMap (OSM) dataset.

**POI types**	**Categories in the OSM dataset**	**Count**	**Percentage**
C	Restaurants, beverages, malls, markets, stores, various shops, greengrocers, hairdressers, vendors, cinemas, car dealerships, car rentals, etc.	8,997	56.22%
M	Chemist, clinic, dentist, doctor, hospital, optician, pharmacy, veterinary, etc.	630	3.94%
E	College, kindergarten, library, playground, school, university, etc.	357	2.23%
P	Governmental organization, social group, communal facilities, financial facilities, convenience, camera surveillance, etc.	5,331	33.31%
R	Scenic spot, park, open square, tourist attraction, theater, viewpoint, etc.	688	4.30%

C, commercial; M, medical; E, education; P, public service; R, recreation.

### 3.3. Micro-scale factors of urban built environment

**Figure 5:**
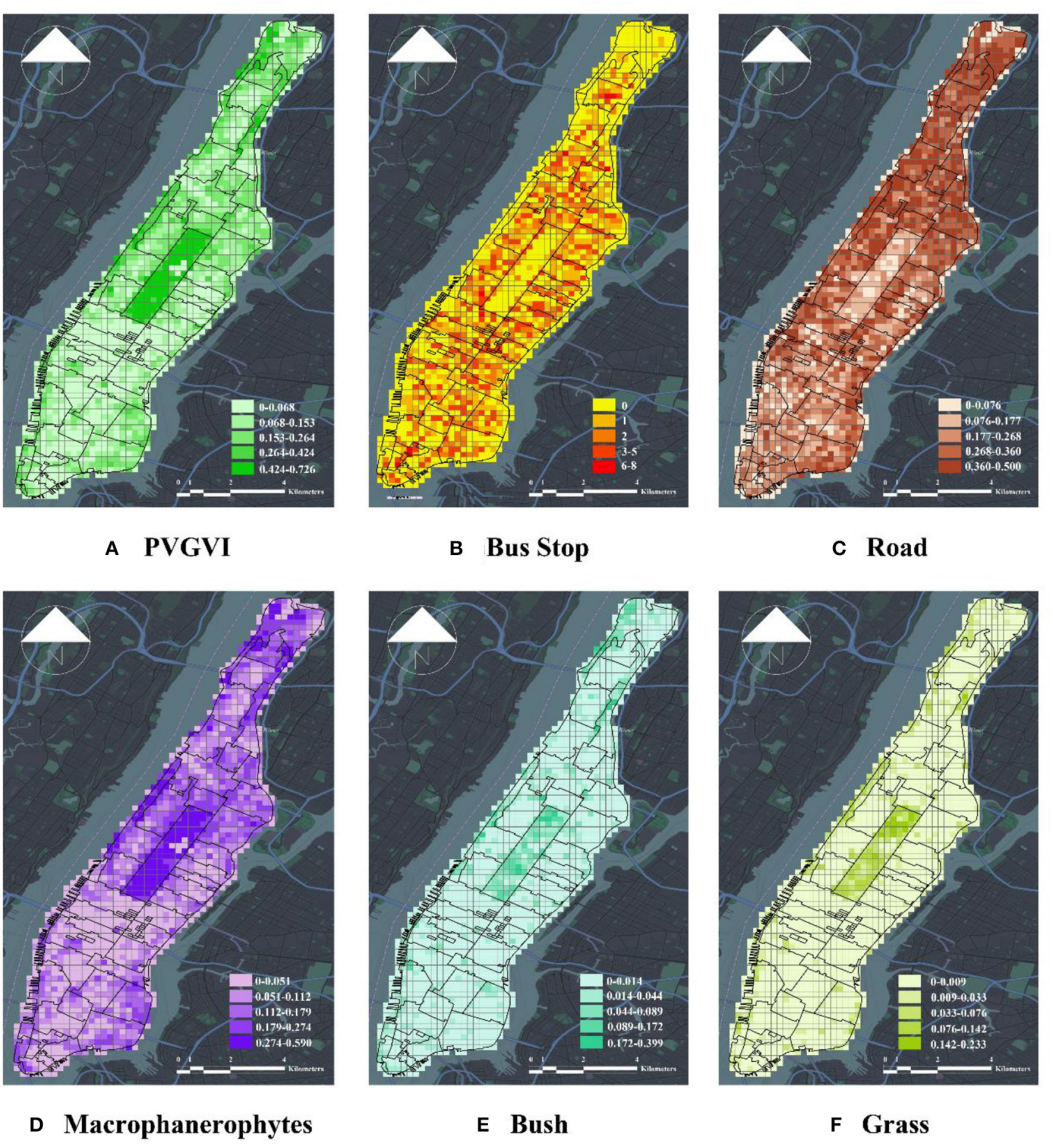
Visualization of micro-scale factors of urban built environment. **(A)** PVGVI, **(B)** bus stop, **(C)** road, **(D)** macrophanerophytes, **(E)** bush, and **(F)** grass.

In this study, micro-level urban built environment specifically refers to the direct perception of the features of the urban landscape by pedestrians. Many studies demonstrate that computer vision combined with panoramic urban streetscape images can extract the features of the urban built environment and evaluate the urban built environment at the street level. This tendency proves that computer vision has gradually entered the scope of urban research. The current study selects the network model open-sourced by Chen et al. (43) in 2018, which pertains to a semantic segmentation network based on the DeepLabV3+ neural network model. The study made this selection for two reasons. The first is that the DeepLabV3+ neural network model is the latest version in the DeepLab series, which modifies VGG16 to introduce null convolution in DeepLabV1. The Atrous Spatial Pyramid Pooling (ASPP) model is designed in DeepLabV2; DeepLabV3 combines. The model proved its accuracy by outperforming mainstream deep learning algorithms (such as SegNet and PSPNet) in performance evaluation competitions such as the PascalVOC and Cityscapes benchmark tests in 2012. The second is that DeepLabV3+ features a better recognition effect compared with other mainstream deep learning models in the interpretation of urban scenes. The reason is that the model is designed for analyzing urban scenes, such that it exhibits certain advantages compared with those of other models when recognizing green structures in urban streets. The third is that the model uses DeepLabV3 as an encoder to generate the features of arbitrary dimensions using Atrous Convolution and adopts the ASPP strategy to use multiple effective sites with upsampling to achieve multiscale feature extraction. Moreover, it uses a cascade decoder to recover boundary detail information. Depthwise Separable Convolution is also used to reduce the number of parameters to further improve the accuracy and speed of the segmentation algorithm. This study selects the panoramic green view rate, the green structure of urban streets, buildings, roads, walls, and sky visibility to represent the micro-scale features of the urban built environment (Figure 5).

### 3.4. Statistical analysis

The study conducted a four-step statistical analysis, namely:

- Pearson's correlation analysis of the CCC and CCR data using all data on the macro- and micro-level factors of the urban built environment, respectively.
- Z standardization of two independent variables. The z-score can transform two or more sets of data into unitless z-scores, which renders data standards uniform and, thus, improves data comparability and weakens data interpretation. The z-standardization formula is as follows:

$$\begin{array}{l} Z=\frac{X-\;\mu }{\sigma }, \end{array}$$

Where μ is the mean and σ is the standard deviation.

iii. The existence of a degree of correlation (approximate covariance) between the explanatory variables can also be called multicollinearity. In this case, the results of parameter estimation are no longer valid; thus, the current study uses variance inflation factor (VIF) to test for potential multicollinearity between the macro- and micro-level independent variables (Table 2). VIF is calculated as follows:

$$\begin{array}{l} VIF=\frac{1}{1-R^{2}}, \end{array}$$

Where *R*^2^ denotes goodness-of-fit or the determination coefficient of linear regression and describes the percentage of explanatory variables in the regression equation. The results indicate the absence of covariance for all independent variables, VIF values are <5, and all factors can be included in the linear regression model.

**Table 2:** Summary statistics of all variables in Manhattan, New York, United States (*n* = 1,551).

**Variables (unit)**	**Min**	**Max**	**Mean**	**SD**	**VIF (*Z*-score)**
Dependent variable					
COVID Case Count (CCC) (*N*)	0	27,410	13814.951	6340.799	
COVID Case Rate (CCR) (*N*)	0	54364.28	30825.563	7509.91	
Independent variables					
Macro-scale built environment					
Public service (*N*)	0	97	3.437	6.911	1.296
Education (*N*)	0	5	0.23	0.576	1.029
Commercial (*N*)	0	63	5.801	8.736	1.417
Medical (*N*)	0	18	0.406	1.055	1.287
Recreation (*N*)	0	14	0.444	1.016	1.244
Airports (*N*)	0	1	0.001	0.025	1.001
Bus station (*N*)	0	2	0.008	0.098	1.041
Bus stop (*N*)	0	8	0.932	1.275	1.175
Ferry (*N*)	0	2	0.004	0.072	1.005
Railway (*N*)	0	3	0.102	0.348	1.144
Taxi (*N*)	0	2	0.006	0.088	1.043
POP (*N*)	0	5722.99	1042.108	1033.205	1.167
Micro-scale built environment					
Sky View Factors (SVF) (%)	0	0.279	0.038	0.042	1.224
Building (%)	0	0.45	0.186	0.109	2.245
Road (%)	0	0.504	0.253	0.131	1.999
PVGVI (%)	0	0.529	0.111	0.104	1.791
Wall (%)	0	0.459	0.058	0.077	1.256
Street greening structure					
Macrophanerophytes (%)	0	0.59	0.114	0.098	1.667
Bush (%)	0	0.399	0.016	0.032	1.518
Grass (%)	0	0.234	0.012	0.029	1.496

(i). All VIF values were standardized using z-score; (ii). Min., minimum; Max., maximum; SD, standard deviation; N, number; %, Percentage; (iii). Concerning the problem that the minimum value of the micro-scale factors of the built environment is 0, the reason is that during panoramic streetscape crawling, certain indoor images will be crawled, which leads to the minimum value of certain micro-scale factors at 0. At the same time, model recognition errors were noted, but the sample size is very small, which will not influence the results.

Lastly, data at different levels with various dependent variables were included in the ordinary least squares (OLS) model. Furthermore, the study employed the White and BP tests to verify whether or not heteroscedasticity exists in the data, to test the original hypothesis that there was no heteroscedasticity in the model, to confirm whether or not the results rejected the original hypothesis, and to determine if there was a rejection of the original hypothesis that there was heteroscedasticity. To address these concerns, the study employed the robust regression method.

## 4. Results

### 4.1. Pearson's correlation analysis

This study used Pearson correlation analysis to examine the correlations between CCC and CCR and 12 macro-level urban built environment (Public, Education, Commercial, Medical, Recreation, Airports, Bus Station, Bus Stop, Ferry, Railway, Taxi, and POP) and 8 micro-level urban built environment (i.e., Sky, Building, Road, Wall, Macrophanerophyte, Grass, Bush, and PVGVI) in Manhattan, New York, USA, respectively, using Pearson's correlation coefficient (PCC) to indicate the strength of the correlations.

**Figure 6:**
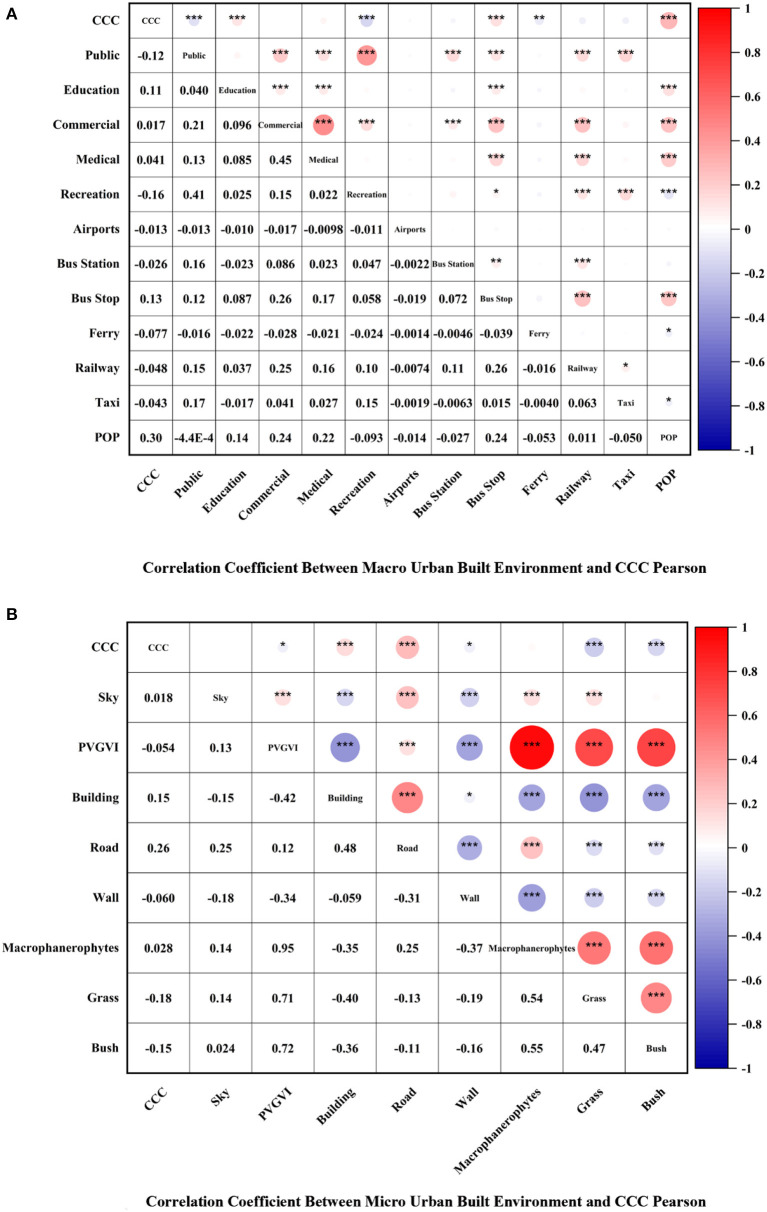
Correlation coefficient between urban built environment and CCC Pearson. **(A)** Correlation coefficient between macro urban built environment and CCC Pearson. **(B)** Correlation coefficient between micro urban built environment and CCC Pearson. *p < 0.05, **p < 0.01, ***p < 0.001.

**Figure 7:**
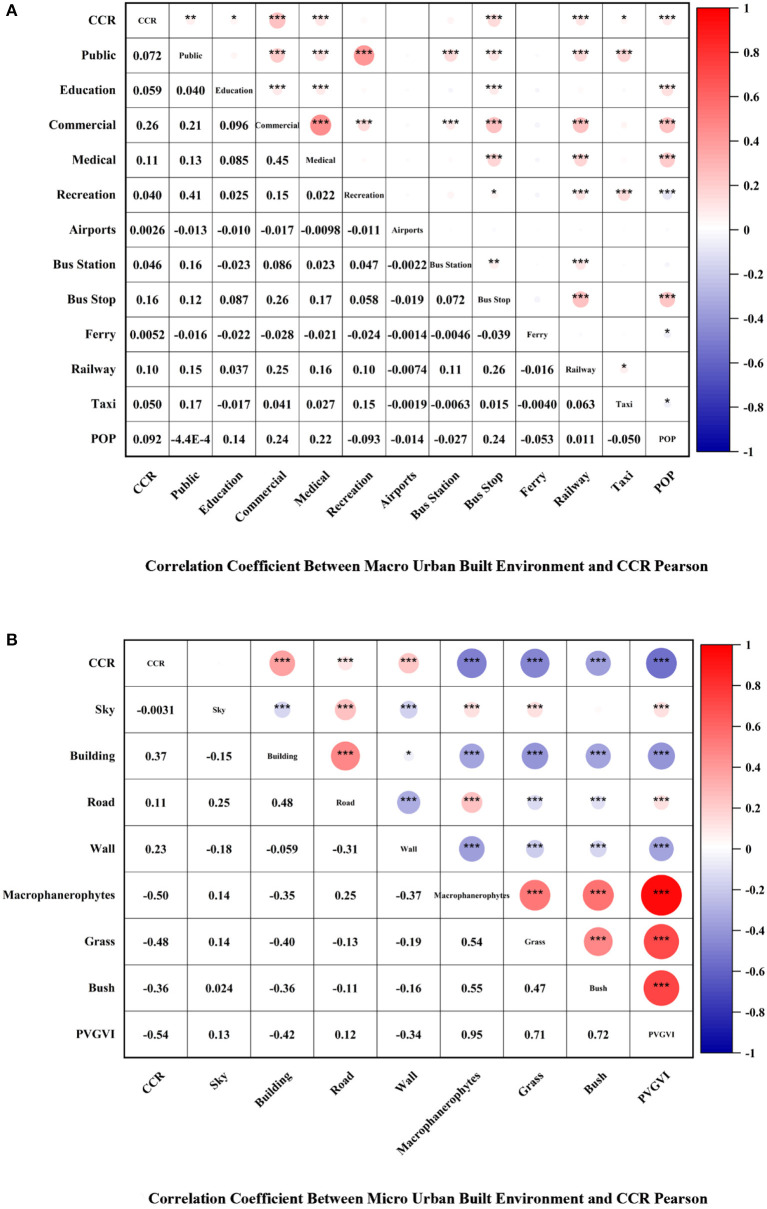
Correlation coefficient between urban built environment and CCR Pearson. **(A)** Correlation coefficient between macro urban built environment and CCR Pearson. **(B)** Correlation coefficient between micro urban built environment and CCR Pearson. *p < 0.05, **p < 0.01, ***p < 0.001.

Figures 6A, 7A depict the relationship between CCC and CCR and macro-level factors, where CCC presents a significant negative correlation with Public (PCC = −0.12, *p* < 0.001), Recreation (PCC = −0.16, *p* < 0.001), and Ferry (PCC = −0.077, *p* < 0.01). Moreover, the study observes a significant negative correlation between Public and Recreation. Both correlations indicate significance at the 0.001 level. Education (PCC = 0.11, *p* < 0.001), Bus Stop (PCC = 0.13, *p* < 0.001), and POP (PCC = 0.30, *p* < 0.001) displayed significant positive correlations with CCC at the 0.001 level of significance. Although the study noted no correlation among Commercial, Medical, Airports, Bus Station, Railway, Tax, and CCC, their PCC values are close to 0, and all *p*-values are >0.05. Figure 4C illustrates that Public, Education, Commercial, Medical, Bus Stop, Railway, Taxi, and POP have significant positive correlations with CCR, where Commercial (PCC = 0.26, *p* < 0.001), Medical (PCC = 0.11, *p* < 0.001), Bus Stop (PCC = 0.16, *p* < 0.001), Railway (PCC = 0.10, *p* < 0.001), and POP (PCC = 0.092, *p* < 0.001) demonstrated showed significance at the 0.001 level, which indicate a significant positive correlation with CCC. Lastly, the study found no correlation among Recreation, Airports, Ferry, and CCR.

Figures 6B, 7B present the relationship of CCC and CCR to micro-level factors, where positive correlations were noted among Building (PCC = 0.15, *p* < 0.001), Road (PCC = 0.26, *p* < 0.001), and CCC, and all of them show. The study found negative correlations among Wall, Grass, Bush, PVGVI, and CCC, where Grass (PCC = −0.18, *p* < 0.001) and Bush (PCC = −0.15, *p* < 0.001) at the 0.001 level of significance, which indicates a significant negative correlation with CCC, whereas no correlation was found between Sky and Macrophanerophytes to CCC. Figure 4D points to a positive correlation among Building, Road, Wall, and CCR at the 0.001 level of significance, which indicates a significant negative correlation with CCR. The correlation between Macrophanerophytes, Grass, Bush, PVGVI, and CCR was all negative at the 0.001 level of significance, among which the PCC value of Macrophanerophytes was −0.5, which extremely exceeded the other variables and indicates a significant negative correlation with CCR.

### 4.2. Robust regression model

The OLS linear regression of CCC and CCR as outcome variables resulted in four models. Macro- and micro-level factors of the urban built environment were separately included as variables in the models to determine the relationship of CCC and CCR to the independent variables at different levels. The equation for OLS linear regression is as follows:


$$\begin{array}{l} Y=X\beta +\;\varepsilon , \end{array}$$

Where *Y* is the dependent variable, *X* denotes the matrix of explanatory variables, β represents the vector of coefficients, and ε is the vector of random error terms.

The variables were included in the OLS model for the White and BP tests. Table 3 presents the results. In the case of heteroscedasticity, the study conducted the White and BP tests to verify the original hypothesis, that is, no heteroscedasticity exists in the model. Table 3 illustrates that both tests reject the original hypothesis at *p* < 0.05, which indicates that heteroscedasticity exists in the model.

**Table 3:** Results of the white and BP tests.

**White heteroscedasticity test**		**BP heteroscedasticity test**	
***X* ^2^**	***P***	***X* ^2^**	***P***
White test and BP test results of CCC and macro urban built environment			
91.844	0.034	24.380	0.018
White test and BP test results of CCC and micro-level urban built environment			
182.923	0.000	120.011	0.000
Results of white test and BP test of CCR and macro urban built environment			
182.923	0.034	120.011	0.000
Results of white test and BP test of CCR and micro-level urban built environment			
435.371	0.000	273.668	0.000

Table 3 suggests that heteroscedasticity exists in the regression data, and the conclusions obtained by the commonly used OLS regression estimation method may be biased because it considers the minimized residual sum of squares as a criterion. Therefore, it also considers anomalous data. In this case in the model regression considered for robust regression analysis (M-estimation), the study uses the Huber robust method with the following formula:


$$\sum_{i=1}^{n}\rho \left(P_{i}^{\frac{1}{2}}\left(a_{i}^{T}X-L\right)\right)=\min\sum_{i=1}^{n}\rho \left(P_{i}^{\frac{1}{2}}\left(a_{i}^{T}X-L\right)\right),$$

where a real function ρ defined in a one-dimensional Euclidean space R is selected for the independent identically distributed equal precision model, such that $a_{i}^{T}$ denotes the row vector of the design matrix; *X* is the extreme value solution; and *P* represents the weight of the corresponding observation or observation error.

Tables 4, 5 depict Models 1 and 2, respectively. The difference between the models is the use of CCC and CCR as the dependent variables, respectively. CCR can be used to illustrate the long-term trend of COVID-19, which could help in analyzing the impact of NPIs on the relationship between the urban built environment and COVID-19. Alternatively, CCC can be used for analyzing the relationship between the impact of a pure urban built environment and COVID-19.

**Table 4:** Model 1: Robust regression results of CCC and urban built environment (*n* = 1,551).

	**Regression coefficient**	**SD**	***t***	***p***	**95% CI**	***R* ^2^**	**Adjusted *R*^2^**	***F***
Constant	0.423	0.02	21.266	0.000^**^	0.384 to 0.462	0.193	0.183	*F*_(20, 1, 530)_ = 18.302, *p* = 0.000
Macro urban built environment								
Public	−0.255	0.088	−2.884	0.004^**^	−0.428 to −0.082			
Education	0.141	0.049	2.885	0.004^**^	0.045 to 0.236			
Commercial	0.116	0.055	2.107	0.035^*^	0.008 to 0.224			
Medical	−0.013	0.107	−0.122	0.903	−0.222 to 0.196			
Recreation	−0.235	0.085	−2.747	0.006^**^	−0.402 to −0.067			
POP	0.297	0.037	8.001	0.000^**^	0.224 to 0.369			
Traffic factors								
Airports	−0.06	0.217	−0.278	0.781	−0.485 to 0.365			
Bus station	0.026	0.115	0.228	0.82	−0.199 to 0.251			
Bus stop	0.138	0.038	3.612	0.000^**^	0.063 to 0.212			
Ferry	−0.363	0.155	−2.351	0.019^*^	−0.666 to −0.060			
Railway	−0.09	0.051	−1.765	0.078	−0.189 to 0.010			
Taxi	0.029	0.128	0.225	0.822	−0.222 to 0.280			
Micro-level urban built environment								
Sky	0.053	0.043	1.237	0.216	0.384 to 0.462			
PVGVI	−0.05	0.022	−2.276	0.023^*^	−0.094 to −0.007			
Building	−0.145	0.037	−3.891	0.000^**^	−0.218 to −0.072			
Road	0.141	0.032	4.374	0.000^**^	0.078 to 0.204			
Wall	−0.115	0.041	−2.8	0.005^**^	−0.196 to −0.035			
Macrophanerophytes	0.213	0.05	4.259	0.000^**^	0.115 to 0.311			
Grass	−0.357	0.058	−6.136	0.000^**^	−0.470 to −0.243			
Bush	−0.198	0.08	−2.481	0.013^*^	−0.355 to −0.042			

Dependent variable: CCC;

* *p* < 0.05,

** *p* < 0.01.

**Table 5:** Model 2: Robust regression results of CCR and urban built environment (*n* = 1,551).

	**Regression coefficient**	**SD**	***t***	***p***	**95% CI**	***R* ^2^**	**Adjust *R*^2^**	***F***
Constant	0.557	0.006	93.445	0.000^**^	0.545 to 0.568	0.283	0.273	*F*_(20, 1, 530)_ = 30.130, *p* = 0.000
Macro urban built environment								
Public	0.07	0.026	2.63	0.009^**^	0.018 to 0.122			
Education	−0.006	0.015	−0.416	0.677	−0.035 to 0.023			
Commercial	0.041	0.017	2.473	0.013^*^	0.008 to 0.073			
Medical	0.006	0.032	0.198	0.843	−0.056 to 0.069			
Recreation	0.044	0.026	1.706	0.088	−0.007 to 0.094			
POP	−0.088	0.011	−7.893	0.000^**^	−0.110 to −0.066			
Traffic factors								
Airports	−0.031	0.065	−0.47	0.639	−0.158 to 0.097			
Bus station	0.028	0.034	0.819	0.413	−0.039 to 0.095			
bus stop	0.012	0.011	1.066	0.287	−0.010 to 0.035			
Ferry	−0.032	0.046	−0.688	0.492	−0.123 to 0.059			
Railway	−0.01	0.015	−0.65	0.516	−0.040 to 0.020			
Taxi	0.049	0.038	1.263	0.207	−0.027 to 0.124			
Micro-level urban built environment								
Sky	0.029	0.013	2.27	0.023^*^	0.004 to 0.055			
PVGVI	−0.063	0.007	−9.421	0.000^**^	−0.076 to −0.050			
Building	0.079	0.011	7.074	0.000^**^	0.057 to 0.101			
Road	0.009	0.01	0.882	0.378	−0.010 to 0.027			
Wall	0.069	0.012	5.574	0.005^**^	0.045 to 0.093			
Macrophanerophytes	−0.047	0.015	−3.123	0.002^**^	−0.076 to −0.017			
Grass	−0.115	0.017	−6.594	0.000^**^	−0.149 to −0.081			
Bush	0.023	0.024	0.952	0.341	−0.024 to 0.070			

Dependent variable: CCR;

* *p* < 0.05,

** *p* < 0.01.

Table 4 depicts the correlation between Model 1 with CCC as the dependent variable and 20 factors of the urban built environment as the independent variables. It uses robust regression analysis (M-estimation) to construct the correlation between the variables of urban built environment and COVID-19. The study finds that the macro-level factors, Education, Commercial, POP, and Bus Stop, exert a significant positive influence on the relationship between the urban built environment and COVID-19. The correlation coefficient of POP was 0.297, which exceeded all other variables. In particular, Commercial is the only factor that exerts a significant positive effect on CCC and CCR as the dependent variables for both regression models. The regression coefficient of Public is −0.255 with a *p*-value of 0.004, which is more significant than the other variables.

The micro-level factors that displayed significant negative effects in the micro-urban built environment were significantly higher; Building, Wall, Grass, Bush, and PVGVI exerted significant negative effects on CCC, where Grass obtained a regression coefficient of −0.357 and a *p*-value of 0.000, which were higher than those of the other variables in the same model in terms of significance and regression coefficient. PVGVI and Grass exhibited a significant negative effect relationship for Models 1 and 2. The regression coefficient for Grass was higher in Model 1 than that in Model 2; however, the significance of both Models is the same (*p*-values = 0.000). Road and Macrophanerophytes exerted a significant positive effect on CCC; both *p*-values were 0.000, which is higher than the other variables in terms of significance, except for Grass, which is equal.

Table 5 presents the results of the robust regression analysis for Model 2 with CCR as the dependent variable and the 20 urban built environment factors as the independent variables. The finding indicates that Public and Commercial show a significant positive relationship with CCR at the macro level, whereas POP indicates a significant negative relationship with CCR. Public and POP produced the opposite results for both models (Model 1: Public: regression coefficient = −0.255, POP: regression coefficient = 0.297; Model 2: Public: regression coefficient = 0.07, POP: regression coefficient = −0.088).

The micro-level factors Sky, Building, and Wall presented a significant positive relationship with CCR, whereas Macrophanerophytes, Grass, and PVGVI pointed to a significant negative relationship with CCR. PVGVI is more significant in Model 2 than it was in Model 1 (Model 1: PVGVIp = 0.023; Model 2: PVGVIp = 0.000). Macrophanerophytes present opposite results in Models 1 and 2 (Model 1: regression coefficient = 0.213; Model 2: regression coefficient = −0.047).

## 5. Discussion

This study investigated the relationship between the factors of the urban built environment and COVID-19 using robust regression analysis (M-estimation) based on solving the heteroscedasticity of the OLS regression model. The study categorized urban built environment into two dimensions, namely, macro and micro, in two urban spatial dimensions, where macro-level factors include variables related to urban traffic, and micro-level factors pertain to urban green structures.

### 5.1. COVID-19 and urban built environment

The study used the relationship between the number (CCC) and incidence (CCR) of confirmed and suspected cases of COVID-19 per 100,000 people in Manhattan, United States, as an entry point for the factors of the urban built environment. However, in the regression analysis with CCR as the dependent variable, POP exhibited a significant negative effect on CCR. In analyzing this entirely contradictory result, the study considered the effect of Commercial, which exerted a significant positive effect on CCC and CCR but with different factors at 0.116 and 0.041, respectively. In other words, residents can obtain necessities in a small area after the outbreak of a potential pandemic, and NPIs are better compared with those in areas with low population density. Residents in these areas must travel long distances to obtain essential resources, where long-distance travel implies increased chances of contact with strangers and COVID-19 infection. In summary: (i) high population density increases the likelihood of human contact, which facilitates the spread of the virus. However, with the implementation of NPIs, residents could only move within a small area; thus, the virus could not spread among areas. (ii) Areas with high-density populations typically have relatively well-developed infrastructure to provide convenient and timely treatment for residents, which, thereby, inhibits the spread of NPI (26, 29). In particular, under strict NPIs, the outdoor activities of residents are restricted, which effectively inhibits the spread of the virus in high-density areas (24). However, at the CCC level, the government for areas with high population density and high commercial activities performs better in terms of pandemic control and detection than did areas with low population density with a higher detection rate than that of areas with low population density. This finding results in a higher number of confirmed and suspected cases compared with those of areas with low population density. At the same time, control and control efforts are correspondingly lower due to the lower population density, which results in an increased number of cases without data. The situation of no data collection. The number of bus stops tends to be proportional to population density; the higher the population density, the higher the number of bus stops. Essentially, bus stops are places where urban residents are most likely to come into contact with strangers. A high frequency of contact with strangers implies an increased chance of infection. In general, public transportation infrastructure that increases population contact is considered a key factor in the spread of infectious diseases (25). Thus, a range of effective measures should be taken to limit the spread of disease in public transport, including limiting passenger density, increasing the frequency of services, and reserving tickets. Other low-carbon and environmentally friendly active transportation modes, such as walking and bicycling, should also be encouraged.

Similarly, at the public level, the results of CCC and CCR indicate a clear contradiction. From the CCR level, the higher the use of public facilities, the higher the probability of exposure to unfamiliar environments. Thus, the virus is likely to spread through public facilities before the introduction of corresponding NPIs. The number of public facilities in areas with high population density far exceeds that in areas with low population density, such that corresponding transmission rates and probability of transmission are also higher. On the contrary, at the CCC level, the frequency of the use of public facilities is suppressed due to NPIs, and residents will voluntarily reduce their frequency of use of public facilities when they are aware of a potential pandemic. This scenario indirectly leads to a negative correlation between CCC and the Public with a regression index of −0.235 over other factors. Schools tend to be places where pedestrian traffic is high, no less than in commercial areas, and the interaction between students and teachers may accelerate the spread of the virus. When students are infected with the virus at school, NCPI can easily infect family members through parent–child interaction, and the spread of the virus within colleges and universities is typically difficult to reasonably control. As the implementation of NPIs led to school closure, the correlation between schools and CCR became negligible; instead, many schools were requisitioned for the isolation of patients, which exerted a positive effect on the control of the outbreak after lockdowns. Alfano et al. (44) demonstrated that the premature opening of schools increased the number of COVID-19 cases in Italy. This result suggests that during an outbreak, the government should implement strict NPIs in schools while ensuring equity in education.

The higher the PVGVI, the farther away from the city center, the lower the population density, and the less space and medium for virus transmission and corresponding inhibitory effects on virus transmission.

Macrophanerophytes exerted a significant positive effect on CCC, whereas Bush and Grass exerted a significant negative effect on CCC when analyzed from the perspective of the green structure of urban streets. The reason for this phenomenon may be that in densely populated areas with developed commercial activities, the green structure is relatively homogeneous and shows a single-tree state. Conversely, areas with a rich green structure have correspondingly low population density and more homogeneous commercial activities, which can be analyzed in combination with macro-level POP and Commercial.

### 5.2. Research values

A series of recommendations for the results of the study have the following applications: (i) they can be applied at the level of prevention of widespread spread of COVID-19 in cities to minimize the risk of infection and the rate of virus transmission among urban residents by exploring the mechanisms of influence of the built environment and COVID-19. Effective control of virus transmission was achieved at the early stage of the outbreak. (ii) Based on the results of the study, government officials and policy makers can better formulate more reasonable NPI policies to prevent widespread infection and cross-infection and reduce the risk of infection among urban residents, while ensuring the wellbeing, health and comfort of urban residents. (iii) The study uses Google Street View panoramic street view images to extract and quantify urban micro built environment factors from the macro built environment and the micro built environment, respectively, to explore the impact of COVID-19 at the urban street level, and the results provide a data base for future urban renewal. This enables cities to play a more important role in facing the trend of COVID-19 epidemic normalization.

### 5.3. Research limitations

This study has its limitations. First, the data published by NYC Health are divided according to the MODZCTA, where individual buildings are designated unique zip codes in several instances. This tendency can exert a confounding effect on the data, and although the study screened a few of the confounding factors at certain levels, this data-level confounding continues to exist. Moreover, although the study sample was expanded according to fishnet divisions, the original sample only comprises 45 areas, which is not representative of all areas in the United States. Second, other demographic data for Manhattan were not available or could not be specifically mapped within each study area, such as household income structure, demographics, gender, underlying disease status, occupation, and ethnic composition, which have been noted in previous studies to be associated with 2019 coronavirus disease transmission. At the same time, within the time point of the COVID-19 pandemic, the lives of residents were frequently restricted by various NPIs, which resulted in extremely complex and confusing life activities and social relationships. Thus, the study selected only 20 variables, which indicates the exclusion of other potential variables such as the density of foot traffic in the region. Previous studies demonstrated that individual behaviors exerted an effect on the spread of COVID-19; however, such variables are statistically unavailable, relatively difficult to obtain, and more difficult to collect in the field due to various policy restrictions imposed by NPIs. The absence of such variables may have led to certain anomalies in the results of the study. Moreover, the effect of spatial autocorrelation cannot be avoided despite the multilevel and multidimensional considerations. Thus, future studies should consider additional aspects and potential variables to explore the relationship between the factors of the urban built environment and COVID-19. This, data on COVID-19 published by NYC Health provided substantial support to various urban studies on COVID-19. However, the published information on the number of cases is, in fact, incomplete due to the lack of statistical data on the number of cases due to the current pandemic policy implemented in the United States. Thus, certain individuals contracted COVID-19 but displayed no symptoms (asymptomatic) due to the lack of assurance of the detection rates of COVID-19 in the population. Moreover, individuals with the infection were not sampled for nucleic acids; thus, they remained unaware of their COVID-19 infection, which rendered their network and range of activities and transmission of the virus virtually uncontrollable and unavoidable. Possible non-linear effects of the variables in this study. The starting point of the robust regression is still based on the processing method of linear data, but the principle of adopting the method should be considered when processing the experimental data, and if the data have non-linear effects, the experimental data can be made permutation substitution so that they are transformed into a linear functional relationship for the test. In future studies, we will apply multiscale geographically weighted regression models with added potential factors to calibrate the existing models for further accuracy in the analysis given that more data are available at the city level.

## 6. Conclusion

The study draws preliminary conclusions on the relationship between the urban built environment and COVID-19 transmission, which focused on the relationship between CCC and CCR as the independent variables and the influence of the urban built environment. The correlation between the urban built environment and COVID-19 transmission was determined using Robust regression analysis (M-estimation). The major findings are summarized as follows:

- Education, Commercial, POP, and Bus Stop exerted a significant positive relationship with CCC at the macro level. Public and Commercial displayed a significant positive relationship with CCR. Public and Recreation have a significant negative relationship with CCC. POP has a significant negative relationship with CCR.
- Macrophanerophytes, Grass, and PVGVI have a significant negative effect on CCR. Road and Macrophanerophytes have a significant positive effect on CCC. Sky, Building, and Wall have a significant positive effect on CCR.
- Medical, Airports, Bus Station, Railway, and Taxi do not exert any influence on the relationship between CCC and CCR at the macro- and micro-levels of the urban built environment.

The current COVID-19 situation remains severe, and predicting the direction of the pandemic is difficult. To cope with more severe pandemic situations, this study provides several recommendations for urban built environments in the context of its results. First, the government should provide easy access to essential resources for urban residents within a controlled range, reduce the frequency of long-distance travel, save travel costs, reduce unnecessary human contact, and control the medium of transmission to reduce the speed and efficiency of the virus transmission. Second, for areas with high population density and commercial activities, strict NPIs should be implemented, such that if a potential outbreak occurs, then the area can quickly and adequately mobilize favorable resources to effectively control the outbreak. Third, the frequency of use of public facilities should be controlled. Although urban public transportation is an important part of the future low-carbon city, it continues to play an important role in the spread of the virus at this stage. In addition, the number of passengers should be controlled, their health status should be strictly tested, and safe social distancing should be observed to effectively control the spread of the virus. The government can promote and introduce incentives to encourage residents to use other modes of travel. Fourth, schools or educational settings were found to be at risk during outbreaks of COVID-19 due to their dense population and foot traffic; thus, a series of strong measures should be taken such as distance teaching or a limited number of people in schools.

The recommendations may serve as a reference for solutions for other cities at the level of controlling the transmission and spread of the virus. Meanwhile, the findings may provide valid suggestions for curbing potential outbreaks of respiratory diseases. However, the applicability of the variables is limited and does not reflect the regional economic level, demographic, and other sociodemographic characteristics of the city due to the limitations of this study and the data sources. Therefore, the generalizability of the results should be carefully considered.

## Author's note

The S-G-S-S datasets used in this study can be downloaded and used from our GitHub site (https://github.com/muteisdope/S-G-S-S-Dataset.git), allowing users to modify, upload, and optimize the datasets. We will continue to upload new datasets and optimize the datasets in the future. Our research team based on Python language, Pytorch deep learning framework, DeepLabV3+ neural network used in our research, the code can be downloaded from our GitHub website (https://github.com/muteisdope/Model.git).

## Data availability statement

The datasets presented in this study can be found in online repositories. The names of the repository/repositories and accession number(s) can be found in the article/supplementary material.

## Author contributions

LZ and XH: conceptualization and writing—original draft. LZ and JW: resources. LW and JW: supervision. LW: validation. All authors contributed to the article and approved the submitted version.
